# Metabolomics and transcriptomics analyses provide new insights into the nutritional quality during the endosperm development of different ploidy rice

**DOI:** 10.3389/fpls.2023.1210134

**Published:** 2023-06-20

**Authors:** Lin Xian, Jiaqi Tian, Yanxi Long, Huijin Ma, Min Tian, Xiangdong Liu, Guoying Yin, Lan Wang

**Affiliations:** ^1^ College of Agriculture, South China Agricultural University, Guangzhou, China; ^2^ Guizhou Academy of Tobacco Science, Guiyang, China; ^3^ State Key Laboratory of Agricultural Genomics, BGI-Shenzhen, Shenzhen, China; ^4^ Guangdong Provincial Key Laboratory of Plant Molecular Breeding, College of Agriculture, South China Agricultural University, Guangzhou, China; ^5^ Guangdong Laboratory for Lingnan Modern Agriculture, Guangzhou, China

**Keywords:** rice, different ploidy, metabolome, transcriptome, nutritional quality, lysine level

## Abstract

Autotetraploid rice is developed from diploid rice by doubling the chromosomes, leading to higher nutritional quality. Nevertheless, there is little information about the abundances of different metabolites and their changes during endosperm development in autotetraploid rice. In this research, two different kinds of rice, autotetraploid rice (AJNT-4x) and diploid rice (AJNT-2x), were subjected to experiments at various time points during endosperm development. A total of 422 differential metabolites, were identified by applying a widely used metabolomics technique based on LC-MS/MS. KEGG classification and enrichment analysis showed the differences in metabolites were primarily related to biosynthesis of secondary metabolites, microbial metabolism in diverse environments, biosynthesis of cofactors, and so on. Twenty common differential metabolites were found at three developmental stages of 10, 15 and 20 DAFs, which were considered the key metabolites. To identify the regulatory genes of metabolites, the experimental material was subjected to transcriptome sequencing. The DEGs were mainly enriched in starch and sucrose metabolism at 10 DAF, and in ribosome and biosynthesis of amino acids at 15 DAF, and in biosynthesis of secondary metabolites at 20 DAF. The numbers of enriched pathways and the DEGs gradually increased with endosperm development of rice. The related metabolic pathways of rice nutritional quality are cysteine and methionine metabolism, tryptophan metabolism, lysine biosynthesis and histidine metabolism, and so on. The expression level of the genes regulating lysine content was higher in AJNT-4x than in AJNT-2x. By applying CRISPR/Cas9 gene-editing technology, we identified two novel genes, *OsLC4* and *OsLC3*, negatively regulated lysine content. These findings offer novel insight into dynamic metabolites and genes expression variations during endosperm development of different ploidy rice, which will aid in the creation of rice varieties with better grain nutritional quality.

## Introduction

Rice (*Oryza sativa* L.) is a food crop that is commonly cultivated worldwide, and China is the world’s largest rice producer, with a total output of nearly 212 million tons in 2022. The current situation of stable rice yield and increased rice yield has resulted from three revolutions, namely dwarf rice breeding, hybrid rice breeding and breeding rice with ultra-high yield. While exploring the improvement of rice yield, the nutritional quality and stress resistance of rice must also be increased. In nature, polyploids are extensively present (Van de Peer et al., 2017; Qu et al., 2023). Most angiosperms, most eukaryotes, and crops such as rice have all undergone polyploidization during the course of evolution (Xiong et al., 2022; He et al., 2023). Some beneficial agronomic characteristics of polyploid rice include large grain size, high weight per thousand grains, a strong stem, and high stress tolerance (Guo et al., 2017; He et al., 2022; Liu et al., 2022; Wang et al., 2022c). Unfortunately, various constraints, including low seed-setting rates, have limited the production and use of such rice (Lu et al., 2020). To overcome this limitation, neo-tetraploid rice lines with greater than 80% seed-setting rates and highly fertile tetraploid rice lines with polyploid meiotic stability (PMeS) genes have been developed (Guo et al., 2017; Ku et al., 2022; Wang et al., 2022c). In addition, our research team developed a new type of tetraploid rice (known as ‘neo-tetraploid rice’), including Huaduo1 to Huaduo 5 and Huaduo 8, which showed normal fertility and strong yield heterosis when crossed with low fertility autotetraploid rice lines (Kamara et al., 2021; Yu et al., 2021a; Chen et al., 2022). Following that, a number of academic and practical studies on high-fertility tetraploid rice were conducted, which encouraged the use of polyploid rice (Ghaleb et al., 2020; Yu et al., 2020; Chen et al., 2021; Ku et al., 2022). In addition to causing gigantism, which increases biomass output, polyploidization also alters the nutritional quality of the organism; for instance, the levels of carbohydrates, proteins, vitamins, and alkaloids typically increase (Sabooni and Gharaghani, 2022). However, rice loses some of its nutritious value as a result of polyploidization. When compared to diploid rice, autotetraploid rice has a protein content that is approximately 30% higher, an amino acid content that is 20–30% higher, and an amylose content that is approximately 12% lower (Gan et al., 2021; Singh et al., 2021). According to a recent study, polyploidization affects the biosynthesis, transport, and deposition of glutelin, which increase the glutelin concentration in rice seeds (Gan et al., 2021). These modifications have made rice more nutrient and flavor dense. In the past, conventional chemical techniques have typically been used to examine the changes in nutrients following polyploidization. Nonetheless, metabolomics is quickly becoming recognized as a crucial analytical approach in nutritional studies due to the essential role that metabolism plays in nutrition (Ulaszewska et al., 2019; Shen et al., 2023). By qualitatively and quantitatively examining all of an organism’s metabolites, a technique known as “metabolomics” can be used to investigate dynamic changes in metabolites as well as accumulation patterns and the genetic bases of plant metabolites (Shen et al., 2023; Wang et al., 2023). A revolutionary method called widely targeted metabolomics analysis allows for the simultaneous quantification of over 1000 known and unknown compounds in addition to hundreds of recognized metabolites (Wang et al., 2018; Li and Song, 2019; Waris et al., 2022; Xue et al., 2022). The nutrient analysis of rice is now being performed increasingly with metabonomics. There were 121 metabolites in mature seeds of *indica* and *japonica* rice, and there were significant differences in their relative quantities (Hu et al., 2014). These findings close the gap between the genome and phenome and make it easier to identify the genetic controls over metabolic traits that can be used as a foundation for the future improvement of rice quality through metabolic engineering. They also provide significant insights into metabolic adaptation in rice subgroups. In addition, Wang et al. (2021) used liquid chromatography-tandem mass spectrometry to compare the metabolites of a group of diploid-tetraploid japonica brown rice and a group of diploid-tetraploid indica brown rice. A total of 401 metabolites were found to differ between the two diploid-tetraploid groups, 180 of which showed opposing expression trends while 221 displayed the same trends. This research offers a foundation for employing polyploidization to alter the nutritional content of rice and serves as a novel theoretical guide for developing nutrient-rich rice cultivars.

However, there have been few studies comparing the metabolites and gene expression of diploid and autotetraploid rice, which has led to limited knowledge about endosperm development after flowering. In our previous, we developed an autotetraploid rice variety, AJNT-4x, with an obviously high seed setting rate of 49.94% by self-crossing for 52 generations. We used iTRAQ-based quantitative glutelin proteomic analysis to identify the physiological metabolism process during endosperm development in terms of protein (Xian et al., 2021). Nevertheless, metabolomics studies on autotetraploid rice are not well developed. AJNT-4x with higher seed setting was studied further at 10, 15, and 20 DAF to reveal the molecular mechanism by coexpression analysis of metabolomics and transcriptomics data. In this research, AJNT-4x and its diploid counterpart AJNT-2x were further researched to identify differentially expressed genes (DEGs) and differential metabolites in different developmental stages. The combination of these phenotypic and molecular findings advances knowledge of biological processes, particularly the control of metabolism and gene expression during the growth and maturation of rice endosperm. Additionally, more studies will be performed on DEGs to clarify the molecular mechanism underlying metabolite accumulation in autotetraploid rice.

## Materials and methods

### Plant materials

On the farm of South China Agricultural University, rice lines (*O. sativa* L. sp. Aijiaonante (AJNT)-2x and AJNT-4x after self-crossing for 54 generations) were produced in the early and late seasons of 2021. Each of the test plots had ten rows planted with a row spacing of 18 to 21 cm. The grains were marked at the beginning of blooming, collected 10, 15, and 20 days after flowering (DAF), and then kept in a freezer at -80°C.

### Metabolite extraction

A total of 100 mg of the sample were weighed into a 2 mL EP tube, 1 mL of 70% methanol aqueous solution and two small steel balls were added, the sample was processed in tissue grinder (50 Hz, 5 min), and the product was refrigerated at 4 degrees overnight (more than 8 h). The samples were removed the following day and centrifuged for 10 minutes at 4°C and 13000 rpm with the supernatant collected, and then the filtered samples were put in a sample vial for LC-MS analysis.

### Ultra-performance liquid chromatography-multi reaction monitor quantitative analysis

The separation and quantitative measurement of metabolites were carried out using a tandem QTRAP6500 Plus high-sensitivity mass spectrometer (SCIEX, USA) and UPLC instrument (Waters, USA). A Waters UPLC HSS T3 (model: 1.8 um*2.1*100 mm) is the chromatography column was utilized. The mobile phases were an aqueous solution containing 0.1% formic acid (solution A) and 100% acetonitrile containing 0.1% formic acid (solution B). The following gradient was used for elution: 0-2 min, 5% solution B; 2-22 min, 5%-95% solution B; 22-27 min, 95% solution B; and 27.1-30 min, 5% solution B. The flow rate was 0.3 mL/min, and the column temperature was 40°C. For the QTRAP 6500 Plus equipped with the EST Turbo Metabolite Spray Interface, the metabolite source parameters were set as follows: metabolite source temperature, 500°C; metabolite spray voltage (IS), 5500 V (positive mode) or -5500 V (negative mode)); and metabolite source gas I (GS1), gas II (GS 2) and curtain gas (CUR) set to 40, 40 and 25 psi, respectively. The MRM technique was carried out in MRM mode, which included the MRM parent-daughter metabolite pair information, collision energy (CE) and declustering potential (DP) and RT (Retention time) of the target metabolite.

According to the RT of metabolite detection, the metabolic logistics intensity of metabolite target separation detection. Each color-coded peak of the mass spectrum represents one metabolite detected. The characteristic metabolites of each substance were screened by the triple four-pole, and the signal response intensity of the characteristic metabolites was obtained in the mass spectrometer. The peak Area of each chromatographic peak was calculated by integrating the mass spectrometer data with MultiQuant software. The peak area of each chromatographic peak represented the relative content of the corresponding substance. Finally, the peak areas of all chromatographic peaks were derived for subsequent statistical analysis.

### Transcriptome sequencing and analysis

A TRIzol Reagent kit (Invitrogen, Carlsbad, CA) was used to extract total RNA. The concentration and purity were determined by both spectrophotometry (A260, A260/280 respectively. The manufacturer’s protocols were followed while creating the cDNA libraries, which constructed from 500 ng of HMW DNA and fragmented using Covaris sonicator E220, and sheared DNA underwent End repair. The adapters kit instructions to make the adapters ligation and clean it with the supplied DNA Clean Beads. PCR amplification was used for purified adapter-ligated DNA and cleaned-up again by magnetic beads. Then, quality control is carried out, purified PCR products were denaturated and ligated to generate single-strand circular DNA libraries and the DNBSEQ system was utilized to sequence the transcriptomes. Adaptor sequences, low-quality reads, and possible contaminants from chloroplast, mitochondrion, and ribosomal DNA were initially removed from the raw reads.

To identify exons and splice junctions, the clean reads were next compared with the *Oryza sativa* L.spp*. japonica* genomic sequence using TopHat. The expression levels of matching genes in each cDNA collection were calculated and normalized to fragments per kilobase of exon per million fragments mapped. For the analysis of hierarchical gene clusters, Cluster 3.0 was used (de Hoon et al., 2004). Using EBSeq, DEGs were detected in several samples (Leng et al., 2013).

### Bioinformatics analysis

Multivariate statistical analysis and univariate analysis were used to screen different metabolites between groups. Change multiple analysis and T test were performed on the data. Fold change (FC) was obtained by variance analysis, and *p* value was obtained by T-test, fold change>1.2 or<0.83, *p* value<0.05.

The three biological replicates of the 6 groups of rice samples were analyzed with the DESeq R program (1.18.0) in transcriptome analysis (Anders and Huber, 2010). DEGs were defined as genes with a *p <*0.05 and an absolute value of log2 (fold change) >1. To find enriched Gene Ontology (GO) categories, the Blast2GO package was applied (Conesa et al., 2005). DEGs were considered substantially enriched for GO keywords with a corrected *p <*0.05. KOBAS software was used to test for the statistical enrichment of DEGs among the Kyoto Encyclopedia of Genes and Genomes (KEGG) pathways (Mao et al., 2005). Using the R package, a coexpression network based on weighted gene coexpression network analysis (WGCNA) was produced (Langfelder and Horvath, 2008), namely, there were four steps: calculation of correlation coefficient between genes, determination of gene modules, co-expression network and correlation between modules and traits, median absolute deviation (MAD) was screened by FPKM value of all genes and the top 75%.

### Gene function analysis by clustered, regularly interspaced, short palindromic repeat gene editing

A CRISPR/Cas9 gene editing construct of *OsLC3* and *OsLC4* was designed as previously described (Wang et al., 2022a). Two targets were designed for *OsLC3* and *OsLC4 via* the CRISPR-GE website (http://skl.scau.edu.cn/). Several rounds of PCR were used to create the expression cassette vector. By means of overlapping PCR, the target sequences were added to the sgRNA expression cassettes. The pYLCRISPR/Cas9 vector based on the Golden Gate system was used to insert the purified PCR products (sgRNA expression cassettes). *Escherichia coli* DH5a competent cells were transformed directly using the ligated products containing the sgRNA expression cassettes. *Agrobacterium tumefaciens* strain EHA105 was used to successfully create CRISPR/Cas9 constructs. Thereafter, investigations using A. tumefaciens-mediated gene transfer were conducted on AJNT-2x backgrounds.

### Lysine level analysis

The content of 13 amino acids (including lysine) of grains in AJNT-2x and AJNT-4x was determined using the fully automatic amino acid analyzer of German Sykam (S-433D), referring to the method of Zhou et al. (2016).

The shells and embryos of rice seeds are removed, and ground into powder in a mortar, and then collected through a pore size of 250 mesh. 0.1g of the sample was added in a hydrolysis tube, then added 12 mL HCl (6M), connecting to a vacuum pump and vacuum it. Hydrolyze at 110°C for 22 hours. After cooling, the hydrolysate was poured into a volumetric flask, and finally was diluted to 30 mL with ddH_2_O. 1 mL of the fixed solution was taken and evaporated to dryness in a 60°C water bath, and then 1 mL of the fixed solution was taken again and evaporated to dryness in a 60°C water bath. Add 3 mL of diluent to dissolve, and then which was filtered through a tetrafluoroethylene membrane, and finally the filter liquor was placed in a brown vial for analysis.

## Results

### Detection of metabolites according to LC–MS/MS

**Figure 1:**
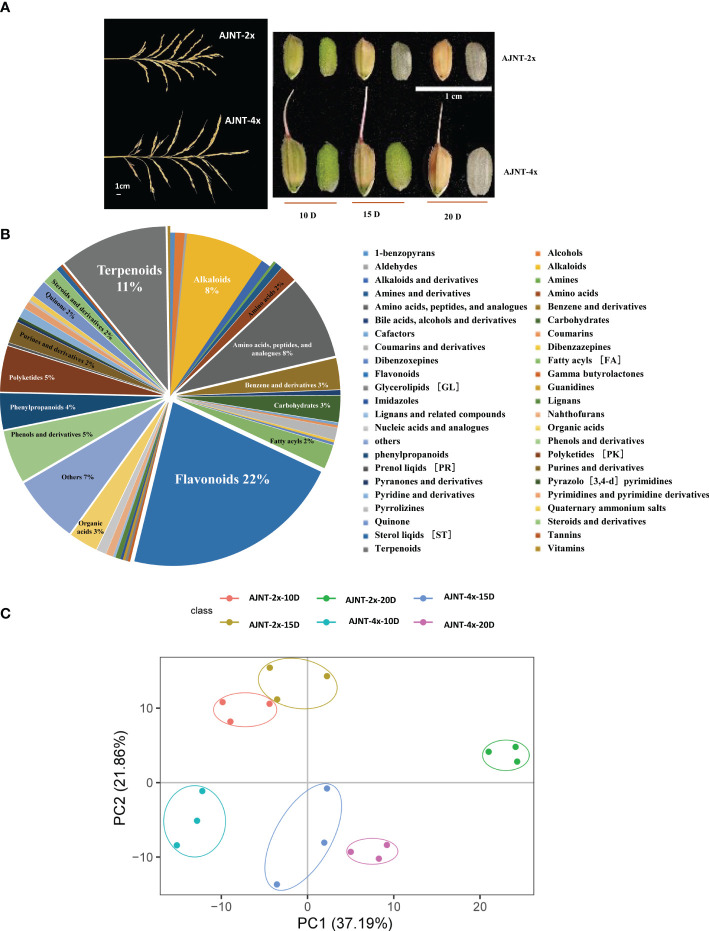
Analysis of metabolites according to LC-MS/MS. **(A)** The spike shape and the grain size of 10 DAF, 15 DAF, and 20 DAF for AJNT-2x and AJNT-4x. **(B)** Classification of the 422 metabolites. **(C)** PCA. The abscissa and the ordinate represent the scores of pc1 and pc2, respectively.

The endosperm of different ploidy rice at 10, 15, and 20 DAF development stages was analyzed by using metabolomics and transcriptomics data (**Figure 1A**). The MRM quantitative software MultiQuant (SCIEX, USA) was used in combination with the extensive targeted metabolic standard database (BGI-Wide Target-Library) independently established by BGI to identify and quantitatively analyze metabolites. The compounds that were found in the samples are represented by the mass spectrometry peaks, each of which represents one metabolite.

QC sample detection curve overlap was extremely high, demonstrating the strong repeatability and dependability of the mass spectrometry data (**Figures S1A, B**). A total of 422 metabolites were detected in the experiment (**Table S1**), including 46 different types of class substances, such as flavonoids, terpenoids, amino acids, peptides and analogs, alkaloids, phenols and derivatives and other metabolites. Among them, flavonoids (21.80%), terpenoids (10.66%), amino acids, peptides and analogs (8.29%), and alkaloids (7.82%) were the most abundant (**Figure 1B**). According to the results of the principal component analysis (PCA), the first principal component (pca1) distinguished between the various treatment groups and explained 37.19% of the variation; the second principal component (pca2) distinguished between the two varieties and explained 21.86% (**Figure 1C**). Our findings demonstrate that the metabolomic profiles of AJNT-4x and AJNT-2x at various phases of development were distinct from one another, and the triplicates of the same sample converged together, demonstrating the high dependability and good reproducibility of the data acquired.

### Metabolic differences between AJNT-4x and AJNT-2x at different developmental stages

**Figure 2:**
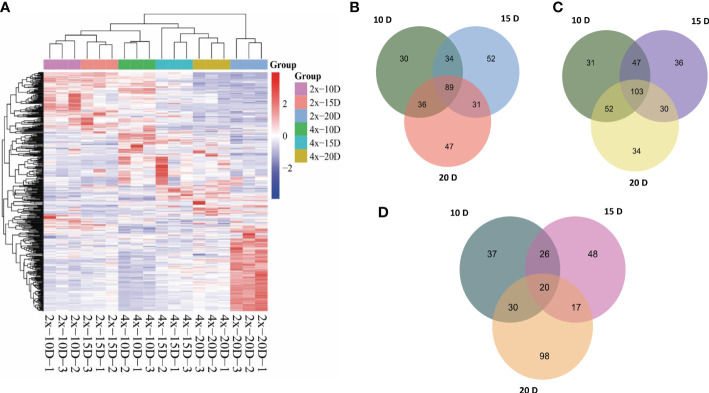
Metabolomic profiles and comparative analysis of AJNT-4x and AJNT-2x. **(A)** Hierarchical cluster analysis (HCA). The color scale shows that the abundance of accumulated is different among samples. The abscissa represents the sample name. **(B)** Upregulation of metabolites in AJNT-4x at 10, 15, and 20 DAF. **(C)** Downregulation of metabolites in AJNT-4x at 10, 15, and 20 DAF. **(D)** Comparison of significantly different metabolites (fold change>1.2 or fold change<0.83, *p* value<0.05) between AJNT-4x and AJNT-2x.

Hierarchical cluster analysis (HCA) was used to examine the accumulation of metabolites between AJNT-4x and AJNT-2x at various developmental phases (**Figure 2A**). There were noticeable differences in abundance between AJNT-4x and AJNT-2x at different developmental stages with regard to the accumulation of these metabolites. Analysis of the differences between AJNT-4x and AJNT-2x showed that the abundances of 189, 206 and 203 metabolites in AJNT-4x were higher than those in AJNT-2x at 10, 15, and 20 DAF, respectively (**Figure 2B**), and 89 kinds of metabolites in AJNT-4x were more abundant than those in AJNT-2x in the three developmental stages. Similarly, the levels of 233, 216 and 219 metabolites in AJNT-4x were lower than those in AJNT-2x at 10, 15, and 20 DAF, respectively. Moreover, 103 kinds of metabolites in AJNT-4x were lower in abundance than those in AJNT-2x in the three developmental stages (**Figure 2C**). The differentially expressed metabolites were examined using the criteria of expression fold changes more than 1.2 with a P value<0.05 (fold change>1.2 or<0.83, P value<0.05) to accurately screen differential metabolites. AJNT-4x and AJNT-2x were compared at 10, 15, and 20 DAF, and 113, 111, and 165 metabolites were discovered (fold change>1.2 or<0.83, P value<0.05), respectively, and 20 metabolites were found in the three developmental stages, which can be considered the key metabolites in different developmental stages (**Figure 2D**).

According to the results of the KEGG classification and enrichment analysis, the differences in metabolites between the compared groups were primarily related to biosynthesis of secondary metabolites, microbial metabolism in diverse environments, biosynthesis of cofactors, ABC transporters, protein digestion and absorption and biosynthesis of alkaloids derived from the shikimate pathway.

### Analysis of differentially expressed genes between AJNT-4x and AJNT-2x in different developmental stages

**Figure 3:**
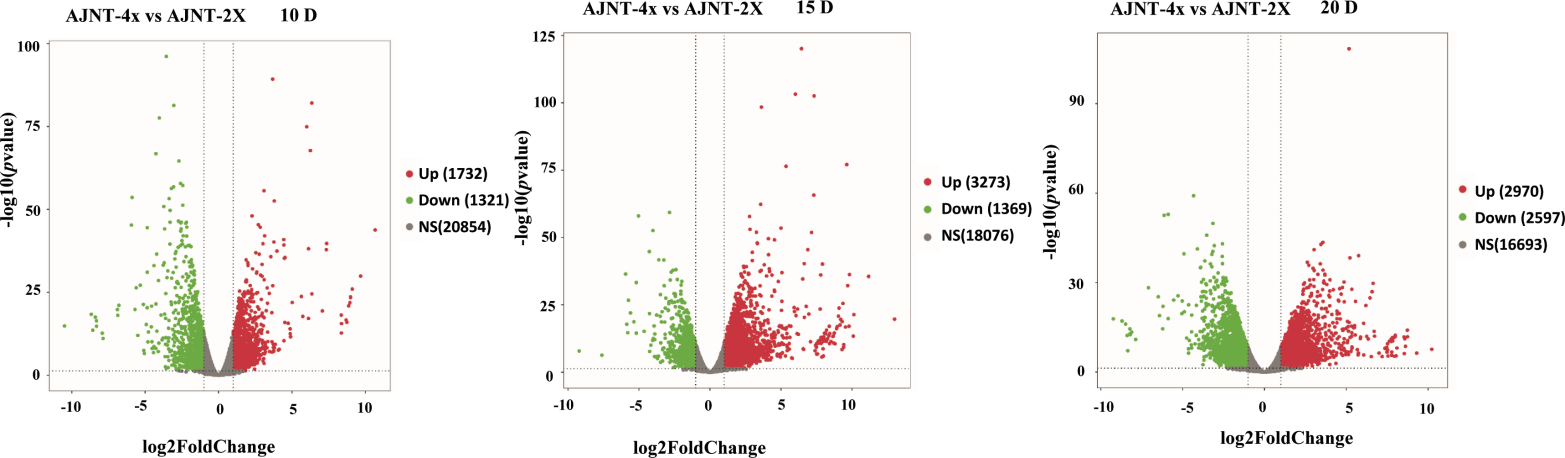
Volcano plot of differentially expressed genes (DEGs). The panel shows AJNT-4x compared with AJNT-2x, from left to right, it’s 10, 15, 20DAF, respectively, red dots represent upregulation and green dots represent downregulation in AJNT-4x (log2-fold change>1 or <-1, *p* value<0.05).

Comparative analysis of AJNT-4x and AJNT-2x at 10, 15 and 20 DAF was conducted, which revealed that 1321, 1369, and 2597 genes were upregulated in AJNT-4x, and 1732, 3273, and 2970 genes were upregulated in AJNT-2x (log2-fold change>1 or <-1, *p* value<0.05) (**Figure 3**). DEGs between AJNT-4x and AJNT-2x at 10, 15 and 20 DAF were compared and annotated by the GO database, and selected for enrichment analysis. At 10 DAF, the main terms were single-organism cellular process in the biological process category, intrinsic to membrane in the cellular component category, and protein binding and hydrolase activity in the molecular function category. At 15 DAF, the enriched biological processes mainly included organic substance metabolic process and primary metabolic process, the main enriched cellular component was intracellular and intracellular part, and the main enriched molecular functions were ion binding and transferase activity. At 20 DAF, the main enriched GO terms cellular metabolic process among biological processes, intracellular part and intracellular for cellular components, and organic cyclic compound binding and heterocyclic compound binding among molecular functions (**Figure S2**).

**Figure 4:**
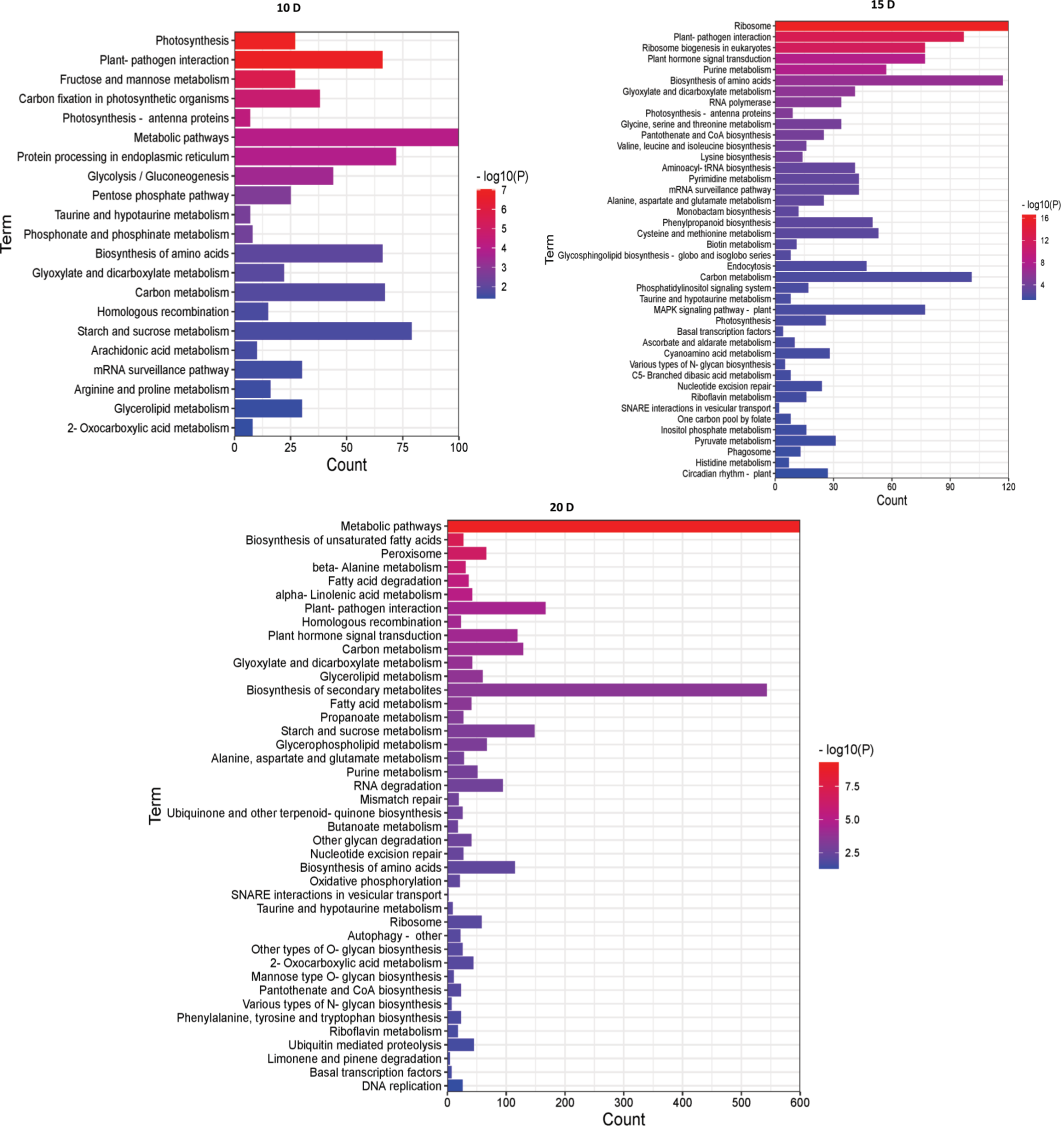
KEGG enrichment analysis of DEGs between AJNT-4x and AJNT-2x. The abscissa represents the number of genes, the ordinate represents the enrichment of pathways, the length of the column represents the number of genes, and the color represents the *p* value.

Then, to examine metabolic processes and possible signaling pathways, these DEGs were examined by KEGG analysis. At 10 DAF, the DEGs were mainly enriched in metabolic pathways and starch and sucrose metabolism. At 15 DAF, the DEGs were mainly enriched in ribosome and biosynthesis of amino acids. At 20 DAF, the DEGs were mainly enriched in metabolic pathways and biosynthesis of secondary metabolites. Interestingly, they were enriched in plant-pathogen interaction, taurine and hypotaurine metabolism, biosynthesis of amino acids, glyoxylate and dicarboxylate metabolism and carbon metabolism (**Figure 4**). Therefore, the number of enriched pathways gradually increased with endosperm development, and the number of DGEs also increased with the development of rice endosperm.

### Analysis of genes related to nutritional quality between AJNT-4x and AJNT-2x at maturity

The nutritional quality of rice is related to the accumulation and metabolism of amino acids. The related metabolic pathways are cysteine and methionine metabolism; tryptophan metabolism; glycine, serine and threonine metabolism; valine, leucine and isoleucine degradation; phenylalanine metabolism; arginine and proline metabolism; lysine degradation; alanine, aspartate and glutamate metabolism; phenylalanine, tyrosine and tryptophan biosynthesis; arginine biosynthesis; tyrosine metabolism; valine, leucine and isoleucine biosynthesis; lysine biosynthesis and histidine metabolism. In addition, the expression of most of these genes was higher in AJNT-2x than in AJNT-4x, but the levels of many amino acids, such as lysine, aspartic acid, and glycine, were higher in AJNT-4x than in AJNT-2x (**Table S2**). There may be a negative correlation between gene expression and some compound accumulation.

### Weighted correlation network analysis between AJNT-4x and AJNT-2x at different developmental stages

**Figure 5:**
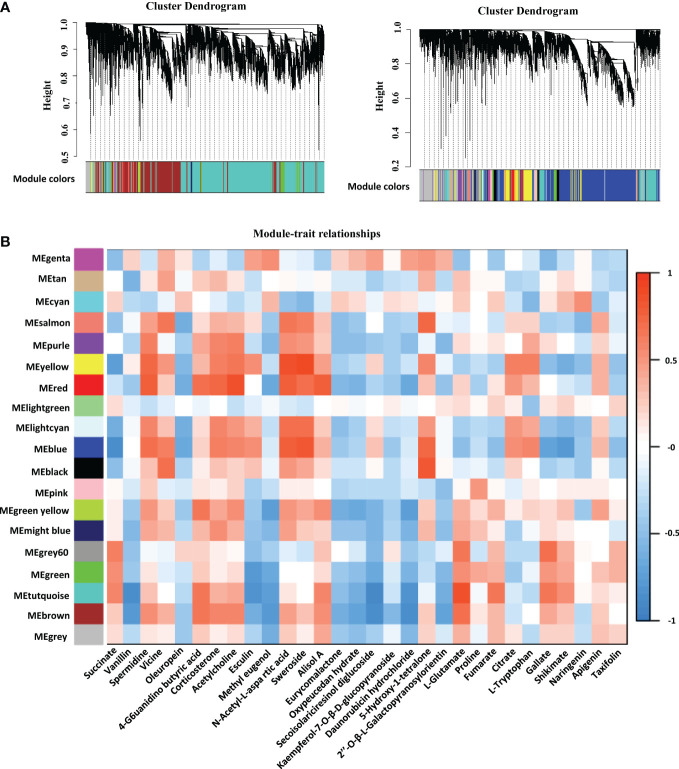
Network analysis dendrogram presenting the modules identified by weighted gene coexpression network analysis (WGCNA). **(A)** All genes are divided into different modules by different functions. **(B)** Module-metabolites weight correlations and corresponding *p* values. The left panel displays the 19 modules. The scale bar on the right illustrates the module-trait correlation from -1 (blue) to 1 (red). The text below represents different metabolites.

We identified 19 WGCNA modules after performing a weighted gene coexpression network analysis (WGCNA) of the FPKM values of all genes and screening those with the top 75% of the median absolute deviation values; nevertheless, the genes in the “MEgray” module were not separated into other modules (**Figure 5A**). To understand the mode-trait correlation, differential metabolites from three stages were selected for analysis, including 20 key metabolites selected from different developmental stages, such as succinate, vanillin, spermidine, vicine, oleuropein, 4-guanidinobutyric acid, corticosterone and other metabolites (**Figure 5B**). The results revealed that spermidine, vicine, 4−guanidinobutyric acid and 9 other metabolites had similar expression patterns, while methyl eugenol, oleuropein, eurycomalactone and 15 other metabolites showed the opposite pattern of expression.

### Molecular function verification of two lysine-related DEGs by CRISPR/Cas9 gene editing technology

**Figure 6:**
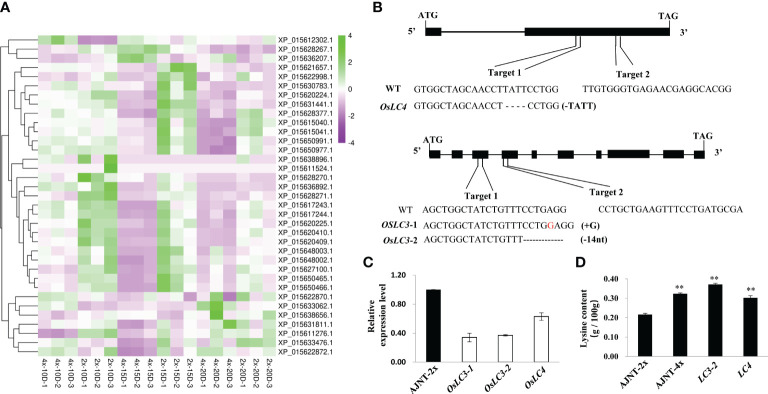
DEGs identified by lysine-related analysis and gene function verification by CRISPR/Cas9 technology. **(A)** DEGs of lysine-related analysis. The abscissa represents the sample name, the ordinate represents the gene name, and the color represents the level of expression. **(B)** *OsLC3* and *OsLC4* generated editing types in the target plant. **(C)** The expression level of the knockout mutant compared with wild-type AJNT-2x in the T_2_ generation, where the two asterisks show a significant difference based on a t test at *p*<0.01. **(D)** The lysine level of the knockout mutant compared with AJNT-2x and AJNT-4x in the T_2_ generation. The two asterisks show a significant difference based on a t test at *p*<0.01.

The nutritional quality of rice is not only determined by its protein content, but also related to the composition and abundance of amino acids, especially the abundance of essential amino acids. Lysine is the first restrictive essential amino acid in rice seeds, and is the main source of nutrition for humans (Yang et al., 2018). Surprisingly, 39 genes related to lysine synthesis were found in the transcriptome analysis, and only 35 of the genes were expressed (**Figure 6A**). Two downregulation genes unreported previously, *XP_015633476.1* and *XP_015631441.1* (namely, *OsLC4* and *OsLC3*, respectively), were selected to verify their molecular function by CRISPR/Cas9 gene editing technology.

In the T_0_ transgenic lines of *OsLC4*, the targets of four of 38 plants were edited, and the first target generated a frameshift mutation with a 4-nt (TATT) deletion, while the second target did not generate a mutant. In the T_0_ transgenic lines of *OsLC3*, the targets of four of 12 plants were edited, and the first target generated two editing types, *lc3-*1 for a 1-nt (G) insertion, and *lc3-*2 for a 14-nt (CCTGAGGTTTGTTT) deletion, while the second target did not generate a mutant (**Figure 6B**). The target mutantions of the two genes all resulted in frameshift mutations of amino acids. T_0_ mutant plants were then grown in T_1_ lines. Homozygous mutants with free T-DNA were examined for lysine content, and their mRNA expression was also checked simultaneously. In the knockout mutants, the mRNA expression level obviously declined (**Figure 6C**), and the lysine level dramatically increased (**Figure 6D**). These results showed that both *OsLC4* and *OsLC3* negatively regulated lysine abundance, which further verified to the transcriptome data. In the autotetraploid rice AJNT-4x, the expression levels of the two genes *OsLC4* and *OsLC3* were lower than those in the diploid rice AJNT-2x, but the lysine abundance in AJNT-4x was higher than that in AJNT-2x.

## Discussion

### Autotetraploid rice may possess better stress resistance and have greater health and nutritional benefits

Plant phenotypes and nutrition are typically altered as a result of polyploidization. Tetraploid rice seeds frequently expand in size and weight compared with those of diploid rice seeds. The degree, rate, gel consistency, alkali spreading value, and gelatinization temperature are all altered at the same time (Gu et al., 2015; Ku et al., 2022). In tetraploid rice, the protein and amino acid levels rise, but the amylose concentration frequently declines (Wang et al., 2022c). Functional proteins, oils, vitamins, flavonoids, trace minerals, necessary amino acids, and functional and essential proteins are unique nutritional components found in rice (Nam et al., 2018; Yang et al., 2019; Sabooni and Gharaghani, 2022; Zhang et al., 2022). Strong antioxidant properties of flavonoids were demonstrated by their ability to directly scavenge free radicals, inhibit the production of free radicals by the enzyme oxidase, chelate metal ions produced by free radicals, and promote the regeneration of antioxidant enzymes and small-molecular antioxidant factors (Bors and Michel, 2002; Borges-Bubols et al., 2013). In this study, A total of 422 metabolites were detected in the experiment (**Table S1**), including flavonoids, terpenoids, amino acids, peptides and analogs, alkaloids, phenols and derivatives and other metabolites. It was found that the abundance of most flavonoids in autotetraploid rice was higher than that in autodiploid rice during endosperm development. Therefore, autotetraploid rice has stronger antioxidant activity, which can better resist oxidation. The flavonoids include hesperidin, methyl hesperidin, naringenin, neohesperidin, naringenin chalcone and 42 other flavonoids. Hesperidin has anti-inflammatory, antiviral, antibacterial and other effects (Wilmsen et al., 2005; Xiong et al., 2019). Methyl hesperidin is as powerful as vitamin P, which can boost vitamin C’s effects and has potent antiviral and antibacterial properties (Jawien et al., 2017). Naringin can inhibit the infiltration of macrophages into fat cells in a high-fat diet, thus inhibiting obesity (Bharti et al., 2014; Chen et al., 2016). Neohesperidin has anticancer, antioxidant and antiviral effects (Gong et al., 2019; Karim et al., 2021). According to the results of the KEGG enrichment analysis, the difference in metabolites were primarily related to biosynthesis of secondary metabolites, microbial metabolism in diverse environments, biosynthesis of cofactors, ABC transporters, and so on. Much of the accumulation of metabolites is generated in the biosynthetic pathway of secondary metabolites and microbial metabolism can affect the accumulation of metabolites (Wei et al., 2019; Su et al., 2023), and polyploid rice include large grain size, high weight per thousand grains (Liu et al., 2022; Wang et al., 2021; He et al., 2022), which may result in that the autotetraploid rice is more nutritious and edible than the diploid rice.

### Between the endosperm developmental stages of AJNT-4x and AJNT-2x, metabolites change more

The metabolites in seeds are crucial determinants of their nutritional value (Farooq et al., 2010; Zhao et al., 2020a). Hence, metabonomics has been frequently utilized to examine seed nutrients frequently (Oikawa et al., 2008; Zhao et al., 2020b). Researchers have previously compared nutrients including amino acids, proteins, and carbohydrates between diploid and autotetraploid rice using chemical methods (Gu et al., 2015; Wang et al., 2021). However, there are no publications on secondary metabolomics comparisons between diploid and tetraploid rice. Based on LC-MS/MS, 422 metabolites from six groups of diploid-tetraploid rice were discovered in this work at various endosperm developmental stages (**Table S1**). The research showed that the metabolite expression abundances were very different between the diploid and tetraploid groups. L-glutamate is a key substance in histidine metabolism, nitrogen metabolism, D-amino acid metabolism and other metabolic pathways and it decreases with the development of rice endosperm. L-glutamate plays an important role in the ornithine cycle (urea synthesis) pathway (Blachier et al., 2009). The amino group can be removed by glutamate dehydrogenase in mitochondria to provide free ammonia for the synthesis of carbamoyl phosphate (Potel et al., 2009; Adeva et al., 2012). In the ornithine cycle (urea synthesis) pathway, glutamate dehydrogenase in mitochondria removes the amino group of glutamate and provides free ammonia for the synthesis of carbamoyl phosphate (Newsholme et al., 2003; Smith and Stanley, 2008). Glutamic oxalate transaminase in the cytoplasm transfers the amino group of glutamic acid to oxaloacetic acid, which then forms aspartic acid through the ornithine cycle, and glutamic acid indirectly provides a second amino group for the cycle (Fernández and Zúñiga, 2006).

Therefore, we speculated that with the development of rice endosperm, L-glutamate could provide increasingly less free ammonia, and the circulation rate of ornithine decreased. L-proline is one of the amino acids that make up human protein, and it is also an ideal osmotic regulating substance that can be used as a membrane and enzyme protective substance and free radical scavenger (Liang et al., 2013; Meena et al., 2019). The growth of plants under osmotic stress plays a protective role, and L-proline also plays a regulatory role in the osmotic balance of the cytoplasm for the accumulation of another important osmotic regulatory substance in the vacuole of potassium ion organisms (Parvaiz and Satyawati, 2008; Takagi, 2008).

**Figure 7:**
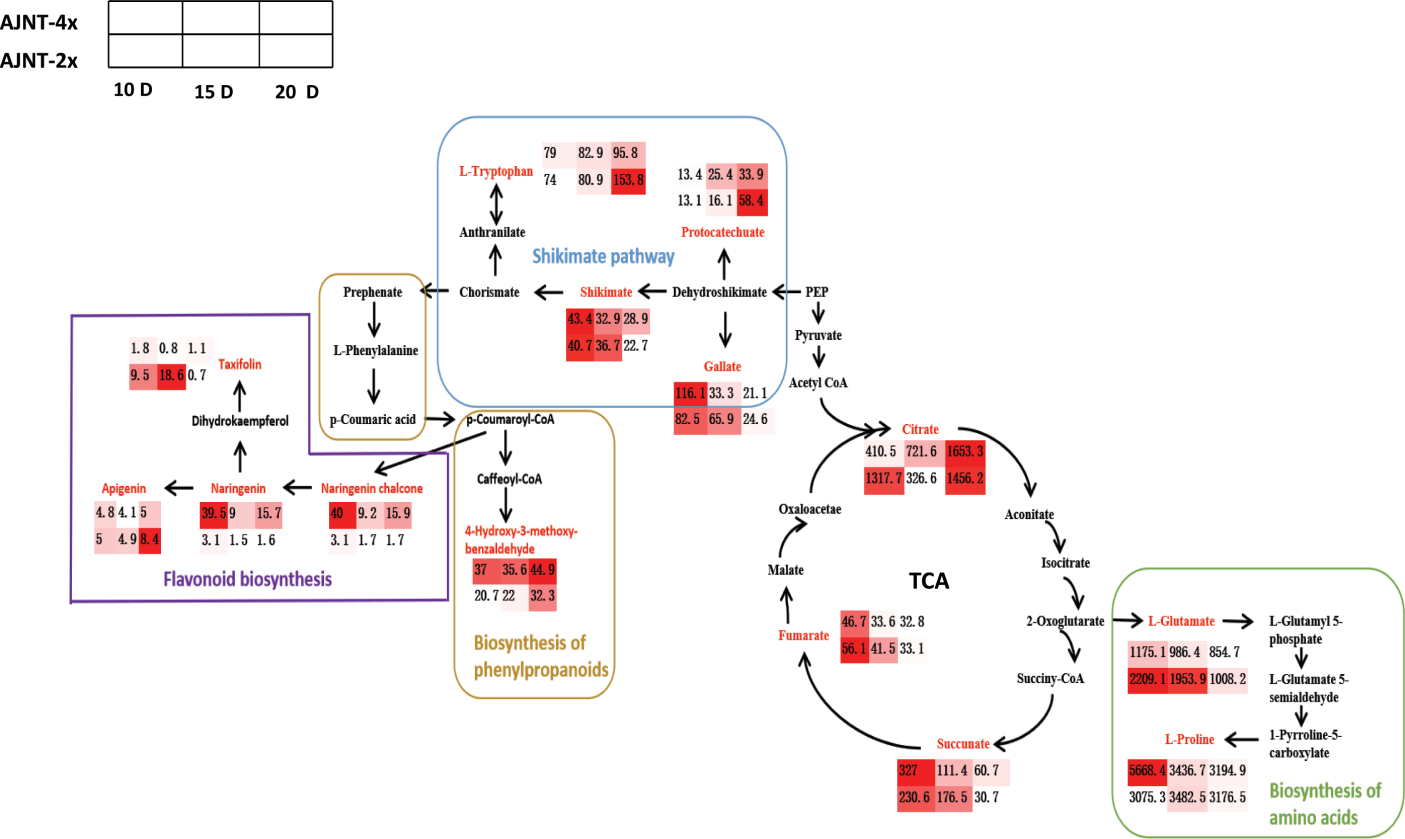
Screening for maps of metabolic pathways involving in key differentially expressed metabolites. The pathway map includes mainly flavonoid biosynthesis, shikimate pathway, biosynthesis of phenylpropanoids, biosynthesis of amino acids and TCA pathway; the red color indicates the differentially expressed metabolites screened; the small heat boxes show the changes in the contents of differentially expressed metabolites between AJNT-4x and AJNT-2x at 10, 15, and 20 DAF, respectively.

Interestingly, the level of L-proline in tetraploid rice decreased with the development of rice endosperm, while the content of L-proline in diploid rice increased first and then decreased, which may indicate that the initial and maximum accumulation times of metabolites differed among materials. The TCA cycle is a ubiquitous metabolic pathway in aerobic organisms (Dijkstra et al., 2011; Wang et al., 2022b). Fumarate and succinate decreased with endosperm development, while citrate increased with endosperm development in tetraploid rice and decreased first and then increased in diploid rice (**Figure 7**). According to WGCNA, genes related to succinate and fumarate synthesis were mainly enriched in the ‘MEturquoise’ module, which contained 11,231 genes (**Figure 5B**). The main functions were positive regulation of signaling, PAS complex and AP-type membrane coat adaptor complex (**Figure S3**), which can impact the TCA cycle process.

### Higher amino acid levels in AJNT-4x than in AJNT-2x during the late stage of development and two novel genes involved in the regulation of lysine synthesis

Humans are unable to produce the nine necessary amino acids valine, leucine, isoleucine, phenylalanine, tryptophan, threonine, histidine, methionine, and lysine (Galili et al., 2016; Shi et al., 2022). Valine can help the body grow normally, repair tissues, control blood sugar, and meet energy needs (Hao et al., 2020; Yu et al., 2021b). Threonine can help people feel less tired, encourage growth and development, hold water in the skin, bind to oligosaccharide chains, preserve cell membranes, and increase phospholipid production and fatty acid oxidation in living organisms (Cheng et al., 2019; Tang et al., 2021). Methionine can protect the liver, treat depression and lower blood pressure (Sanderson et al., 2019; Lauinger and Kaiser, 2021). Lysine is the first limiting amino acid in the human body and has very important physiological functions in the human body (Shewry, 2007; Liao et al., 2015). It can participate in the synthesis of skeletal muscle, enzymes and peptide hormones and is one of the ketogenic amino acids. When the body lacks available carbohydrates, lysine can participate in ketone body synthesis and glucose metabolism to maintain acid-base balance in the body (Li et al., 2020). Inadequate lysine intake will block the absorption of other amino acids, thus hindering the effective utilization of rice protein, resulting in the imbalance of dietary structure and nutritional composition of food and ultimately the weakening of human metabolic function and metabolic disorders (Yao et al., 2020). The lysine level of tetraploid and diploid rice at 15 and 20 DAF was detected and found to be higher in tetraploid rice (Xian et al., 2021).

In this study, we found that the content of most essential amino acids in AJNT-4x was higher than that in AJNT-2x, so we assume that the nutritional value of tetraploid rice is higher than that of diploid rice. In addition, combined with transcriptome analysis, 39 genes related to the lysine synthesis pathway were identified, and only 35 of the genes were expressed. The expression levels of most genes were lower in tetraploids than in diploids, and these genes have the potential to reverse regulate lysine synthesis. Two novel genes (*OsLC4* and *OsLC3*) were selected to verify their molecular function by CRISPR/Cas 9 gene editing technology. We found that the target mutations of the two genes resulted in frameshift mutations of amino acids, the mRNA expression level obviously declined, and the lysine level dramatically increased in the knockout mutants. Therefore, the *OsLC4* and *OsLC3* genes both negatively regulate lysine abundance, and we will select more genes related to lysine synthesis for verification in the future.

## Conclusion

In conclusion, a total of 422 metabolites were observed between AJNT-4x and AJNT-2x, and 3053, 4642, and 5567 DGEs were identified at 10, 15 and 20 DAF, respectively. We identified key metabolites participating in the biosynthesis of amino acids and phenylpropanoids and the metabolism of glutathione and inositol phosphate, which provide energy and raw materials for rice polyploids and performed WGCNA to understand the regulatory genes of metabolites. In addition, by using CRISPR/Cas9 gene-editing technology, we found that two novel genes, *OsLC4* and *OsLC3*, negatively regulated lysine abundance. These results provide new insights into dynamic metabolite and gene expression differences during endosperm development in autotetraploid rice, which will aid in the development of rice cultivars with increased yield and improved grain nutritional quality.

## Data availability statement

The datasets generated for this study can be found in the Supplementary material, and China National GeneBank DataBase (CNGBdb) database under the accession number: CNP0004286.

## Author contributions

LX: performing the experiments, analyzing the data, writing-original draft, writing-review and editing. JT: writing-review and editing. YL: performing the experiments. HM: performing the experiments. MT: performing the experiments. XL: developing autotetraploid rice AJNT-4x, writing-review and editing. GY: funding acquisition, writing-review and editing. LW: funding acquisition, designing the research, analyzing the data, writing-review and editing. All authors contributed to the article and approved the submitted version.

## Supplementary material

Supplementary Figure 1 Detection of metabolites according to LC-MS/MS. **(A)** MRM metabolite detection multipeak map (multisubstance extraction ion current spectrum, XIC). Note: The abscissa is the retention time of metabolite detection (retention time, Rt). The ordinate is the ion current intensity of the ion detection (the intensity units are counts per second (cps)). **(B)** Detection of the TIC overlap map by QC sample mass spectrometry.

Supplementary Figure 2 GO enrichment analysis of DEGs between AJNT-4x and AJNT-2x. The ordinate represents the functions of biological process (BP), cellular component (CC) and molecular function (MF), and bubble size represents the number of genes.

Supplementary Figure 3 Enrichment of graph. Note: Four circles from the outside to the inside. The first circle: enriched classification, outside the circle is the scale of gene number. Different colors represent different categories. Second circle: the number of background genes in this category and Q or P values. The more genes there are, the longer the bar, the smaller the value, and the redder the color. Third circle: the total number of foreground genes. Fourth circle: RichFactor value of each classification (the number of foreground genes divided by the number of background genes in the classification). Each small cell of the background auxiliary line represents 0.1.
