# Supplementary figures and images for: Metabolomics and transcriptomics analyses provide new insights into the nutritional quality during the endosperm development of different ploidy rice
Source: Front Plant Sci. 2023 Jun 20;14:1210134. doi: 10.3389/fpls.2023.1210134 (PMC10319422; doi:10.3389/fpls.2023.1210134)

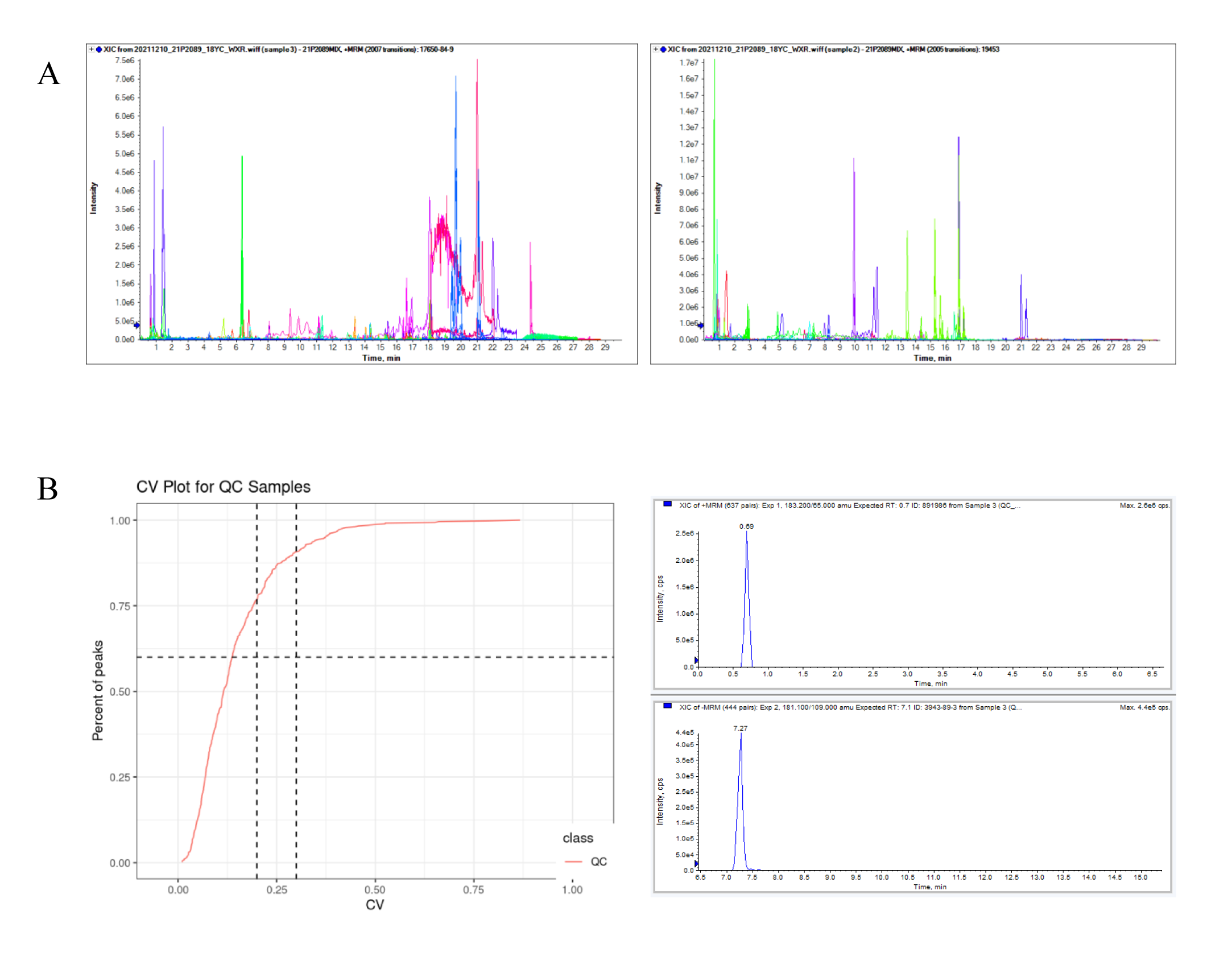

Supplement: Supplementary Figure 1 — Detection of metabolites according to LC-MS/MS. (A) MRM metabolite detection multipeak map (multisubstance extraction ion current spectrum, XIC). Note: The abscissa is the retention time of metabolite detection (retention time, Rt). The ordinate is the ion current intensity of the ion detection (the intensity units are counts per second (cps)). (B) Detection of the TIC overlap map by QC sample mass spectrometry. [file Image_1.tif]

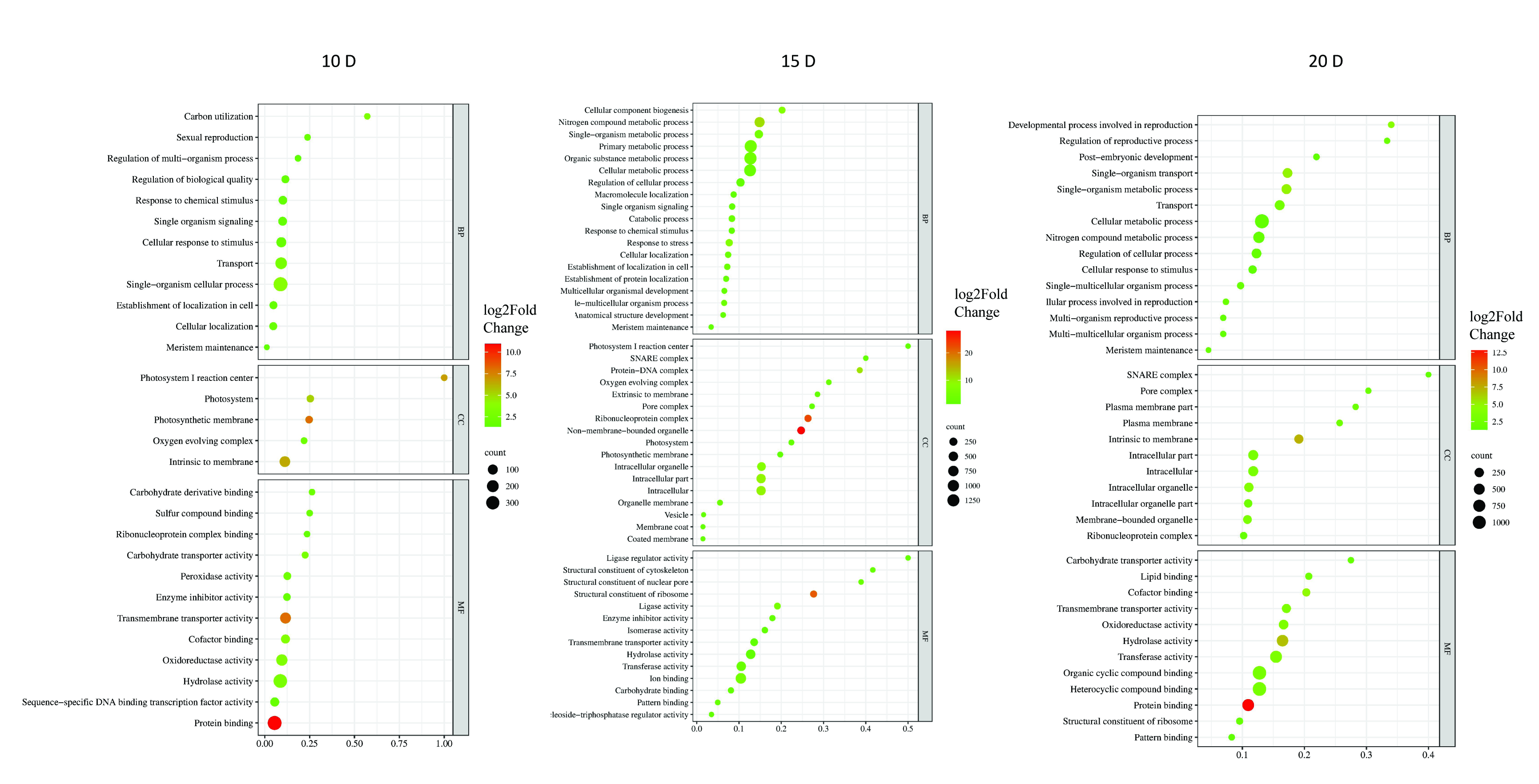

Supplement: Supplementary Figure 2 — GO enrichment analysis of DEGs between AJNT-4x and AJNT-2x. The ordinate represents the functions of biological process (BP), cellular component (CC) and molecular function (MF), and bubble size represents the number of genes. [file Image_2.tif]

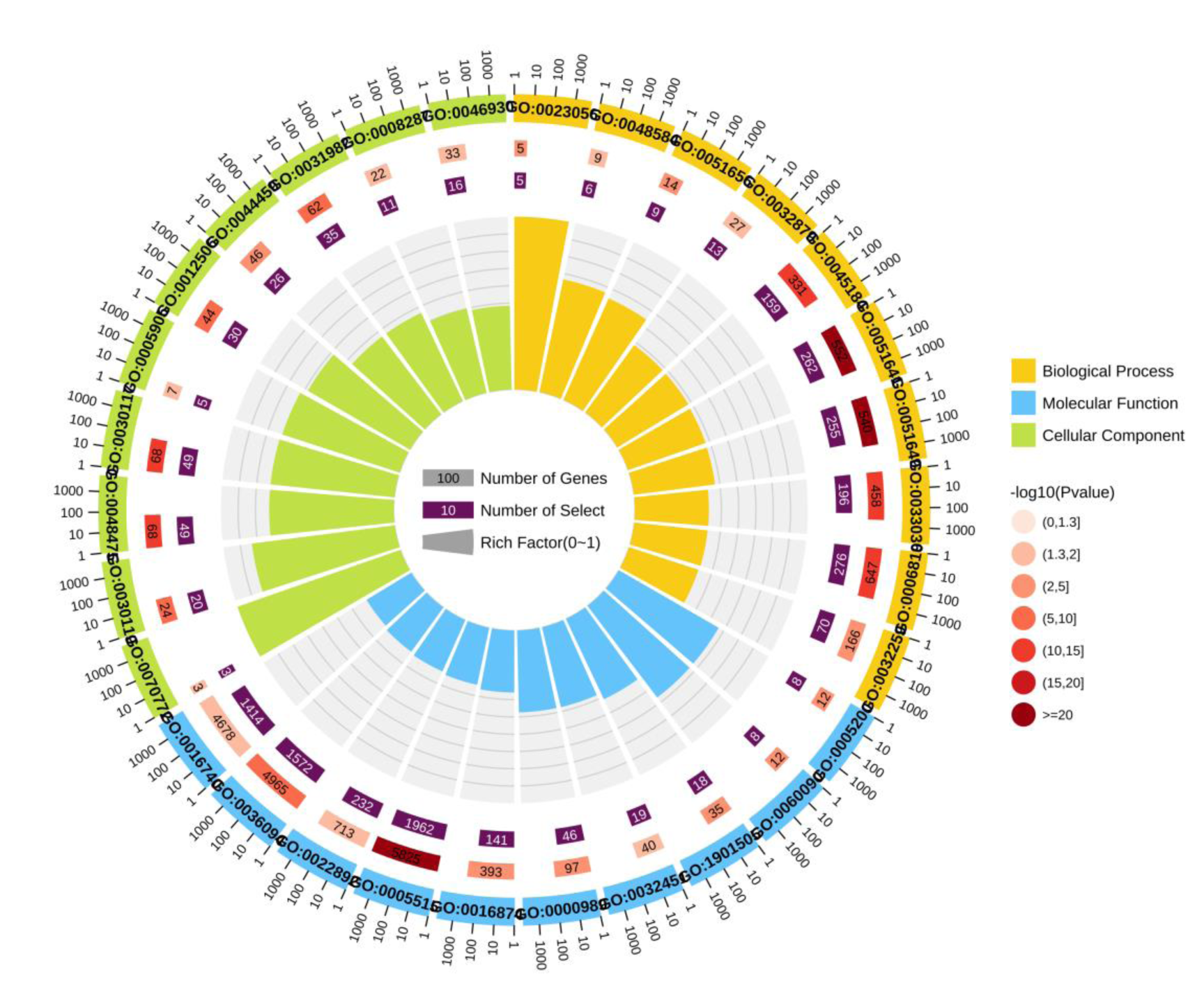

Supplement: Supplementary Figure 3 — Enrichment of graph. Note: Four circles from the outside to the inside. The first circle: enriched classification, outside the circle is the scale of gene number. Different colors represent different categories. Second circle: the number of background genes in this category and Q or P values. The more genes there are, the longer the bar, the smaller the value, and the redder the color. Third circle: the total number of foreground genes. Fourth circle: RichFactor value of each classification (the number of foreground genes divided by the number of background genes in the classification). Each small cell of the background auxiliary line represents 0.1. [file Image_3.tif]
