# Supplementary material for: Metabolomics and transcriptomics analyses provide new insights into the nutritional quality during the endosperm development of different ploidy rice
Source: Front Plant Sci. 2023 Jun 20;14:1210134. doi: 10.3389/fpls.2023.1210134 (PMC10319422; doi:10.3389/fpls.2023.1210134)
Supplement: Supplementary file 5 [file DataSheet_1.pdf]

Table S2 Determination of amino acids in mature periods

Varieties	Aspartic acid	Threonine	Serine	Glycine	Alanine	Valine	Cysteine	Methionine	Isoleucine	Phenylalanine	Lysine	Histidine	Arginine
AJNT-4x	0.96	0.369	0.474	0.454	0.614	0.564	0.11	0.2	0.411	0.499	0.372	0.267	0.725
AJNT-2x	0.822	0.315	0.401	0.404	0.53	0.482	0.1	0.181	0.346	0.425	0.318	0.233	0.631
